# Quality of Life and Complications Among Hemodialysis Patients

**DOI:** 10.7759/cureus.71086

**Published:** 2024-10-08

**Authors:** Mohamed Elsherbiny, Sarah Almuhanna, Reem Alathel, Yara Alzahrani, Fahda Alshathri, Abdulrahman Hussamuldin, Alaa Alherz, Sara Abdalla

**Affiliations:** 1 Anatomy, AlMaarefa University, Riyadh, SAU; 2 Nephrology, AlMaarefa University, Riyadh, SAU; 3 Nephrology, Alexandria University, Alexandria, EGY

**Keywords:** chronic kidney failure, hemodialysis, kidney impairment, quality of life, renal failure

## Abstract

Background

Patients with renal impairment who require frequent hospitalizations or dialysis must undergo the rigorous process known as hemodialysis (HD). Hemodialysis is a complex treatment for people who require frequent hospital or dialysis appointments, typically three times each week. We aim to explore the quality of life of hemodialysis patients and their complications.

Methodology

This case-control study focuses on renal failure patients in Riyadh's hemodialysis units. The case group included 50 hemodialysis patients with 50 healthy individuals medically free in the control group who were the same gender and age as the case group. The comparison was made using a quality of life (QoL) short form 36 questionnaire conducted by a trained physician via Google Forms. The data was obtained in February and March 2023.

Results

The majority of participants were male (68; 68%), aged 40-50 years (42; 42%), 53 (53%) were non-Saudi, 76 (76%) began hemodialysis one year ago, and 30 (30%) developed an infection as a complication. There was a significant statistical relationship between our results and the results of a medical outcome study in the emotional problem and energy/fatigue domains.

Conclusion

In all domains, HD patients performed worse on subscales than healthy people of the same age and socioeconomic status. The average QoL score for HD patients was lower than that of healthy individuals. There was a statistically significant relationship between healthy people and hemodialysis patients in two domains: emotional problems and energy/fatigue levels.

## Introduction

Chronic renal disease is defined as kidney dysfunction or having a glomerular filtration rate (GFR) of less than 60 mL/min/1.73 m^2^ for at least three months. End-stage renal failure is a chronic illness that has a major impact on patients' health-related quality of life, owing to considerable impairment in a variety of daily functioning areas [1]. Long-term dialysis therapy has been linked to a decrease in personal autonomy, increasing reliance on caregivers, disruption of marital, familial, and social relationships, and a reduction or full loss of financial resources. The aforementioned issues had negative consequences for their physical, psychological, economic, and environmental well-being [2]. Functional vascular access is necessary for providing adequate hemodialysis treatment [3]. Hemodialysis is a difficult treatment that necessitates patients' frequent trips to hospitals or dialysis centers, usually three times each week. As a result, it alters patients' normal living routines. In 2014, Saudi Arabia, commonly known as the Kingdom of Saudi Arabia (KSA), registered a total of 14,704 occurrences [4]. End-stage renal disease (ESRD) is the most advanced and final stage of chronic renal disease, in which patients frequently require hemodialysis (HD) to survive. Long-term dialysis therapy frequently causes a variety of issues, including decreased personal autonomy, reliance on caregivers, disturbance of intimate relationships, family dynamics, and social connections, as well as potential financial pressure [5]. The increasing demand for hemodialysis therapy places a significant financial and environmental strain on healthcare systems [6]. The existence of around 13 outpatient dialysis centers in specific localities is fundamentally related to a lower overall quality of life. A cluster selection method was used to choose a sample of end-stage renal disease (ESRD) patients of various ages who were receiving dialysis treatment. The sample was collected from five outpatient hemodialysis centers and two patient dialysis centers. Individuals diagnosed with any type of neurological condition were barred from participating in the study. Given Saudi Arabia's projected population of 14,704 dialysis patients, a sample size of 245 patients was computed with an 80% power level. Before beginning the investigation, a preliminary investigation with a cohort of 30 patients was conducted [7,8]. Assessing the quality of life in hemodialysis patients is critical in controlling the disease and its consequences, as it can have a considerable impact on the patient's prognosis and overall survival [9]. The primary objective of this study is to assess the quality of life in hemodialysis patients and to identify the complications affecting their overall well-being.

## Materials and methods

A case-control study, targeting patients with renal failure who are in a hemodialysis unit in a hospital in the central region of Riyadh city. The case includes 50 hemodialysis patients and the control of 50 healthy individuals medically free with the same gender and age as the case group, the comparison is through a quality of life (QoL) short form 36 (SF-36) questionnaire administered by a trained physician via Google Form. Data were collected in February and March 2023. The SF-36 questionnaire is used to indicate the health status of populations, to help with service planning, and to measure the impact of clinical and social interventions. The 36-item scale measures eight domains of health status: physical functioning (10 items); physical role limitations (four items); bodily pain (two items); general health perceptions (five items); energy/vitality (four items); social functioning (two items); emotional role limitations (three items), and mental health (five items). Demographic data include age, gender, and nationality. A consent form was obtained from the participants to inform them about the possible common complications related to the method in this study: hypotension, infection, blood clot, or others, which they were extracted from hospital records. Descriptive statistics were calculated for all variables of interest. Frequencies and percentages were used to summarize categorical variables; this approach provided a clear overview of the demographic characteristics and health-related behaviors of the study sample. A p-value of less than 0.05 was considered statistically significant. This threshold was used to determine the presence of significant differences. Statistical analyses were conducted using IBM SPSS Statistics version 27.0.1 (IBM Corp, Armonk, NY, US). This software was utilized to perform all descriptive and inferential statistical tests, ensuring accurate and reliable results.

## Results

Out of the total (N=100) participants in this case-control study, 68 (68%) were men and 32 (32%) were women. The majority 42 (42%) were between the ages of 40 and 50. Regarding nationalities, 53 (53%) are not Saudi. Furthermore, 63 (63%) of the participants have a university degree, 78 (78%) of the participants are married, with only 4 (4%) widowed. Table 1 shows the personal information of the participants.

**Table 1:** Personal information

Gender	Frequency	Percent
Male	68	68.0
Female	32	32.0
Age	Frequency	Percent
18-28	6	6.0
29-39	12	12.0
40-50	42	42.0
51 and more	40	40.0
Nationality	Frequency	Percent
Saudi	47	47.0
Non-Saudi	53	53.0
Education Level	Frequency	Percent
Illiterate	10	10.0
Primary School	4	4.0
Middle-High School	23	23.0
University degree	63	63.0
Social Status	Frequency	Percent
Single	12	12.0
Married	78	78.0
Divorced	6	6.0
Widowed	4	4.0
Total	100	100.0

**Figure 1:**
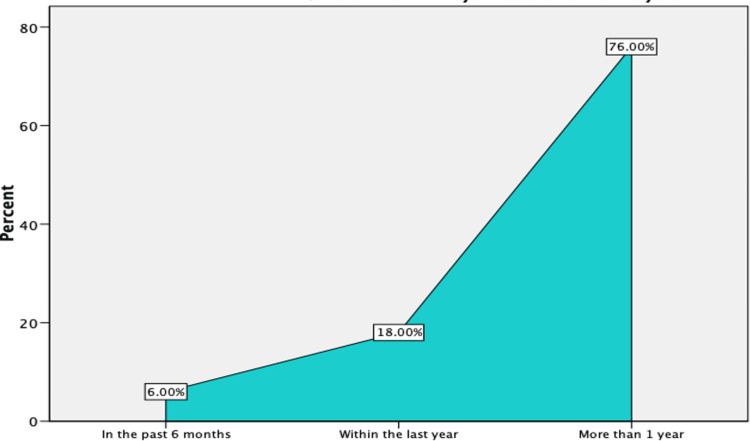
Hemodialysis patients (N=50)

Figure 1 shows that 76% of the hemodialysis patients in this study began their treatment more than a year ago, 18% within the previous year, and only 6% within the last 6 months.

**Figure 2:**
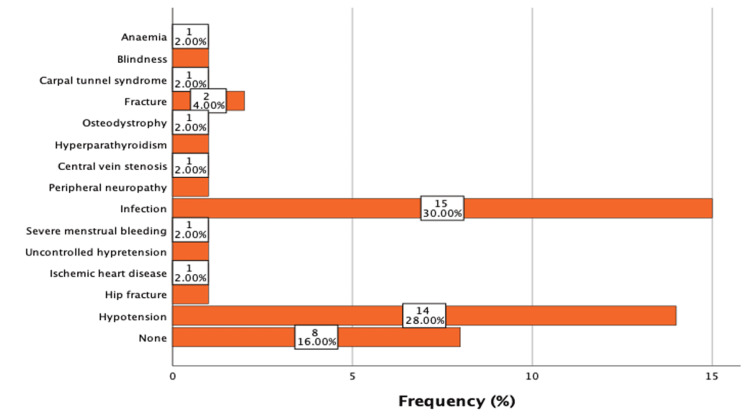
Hemodialysis complications (N=50)

Figure 2 shows that among all the hemodialysis patients in this study, 15 (30%) reported infection as a problem, followed by 14 (28%) with hypotension, and 8 (16%) with no consequence. Following that, two (4%) had a fracture and one (2%) reported each of the following complications: anemia, blindness, carpal tunnel syndrome, osteodystrophy, hyperparathyroidism, central vein stenosis, peripheral neuropathy, severe menstrual bleeding, uncontrolled hypertension, ischemic heart disease, and hip fracture.

Table 2 shows that hemodialysis patients reported poor quality of life in all eight domains, including 37 (74%) in physical function, 41 (82%) in physical health, 27 (54%) in emotional problems, 41 (82%) in energy fatigue, 45 (90%) in emotional well-being, nearly all 46 (92%) in social function, 44 (88%) in pain, and 41 (82%) in general health. In contrast, healthy people reported high quality of life in all domains, including 40 (80%) in physical function, 42 (84%) in physical health, 44 (88%) in emotional problems, 33 (66%) in energy fatigue, 28 (56%) in emotional well-being, 35 (70%) in social function, 34 (68%) in pain, and 38 (76%) in general health.

**Table 2:** Participants responses to the quality of life short form 36 (SF-36) questionnaire

Hemodialysis Patients Response (HD)		Frequency	Percent
Physical Function	Good Quality of Life	13	26.0
	Poor Quality of Life	37	74.0
Physical Health	Good Quality of Life	9	18.0
	Poor Quality of Life	41	82.0
Emotional Problem	Good Quality of Life	23	46.0
	Poor Quality of Life	27	54.0
Energy Fatigue	Good Quality of Life	9	18.0
	Poor Quality of Life	41	82.0
Emotionally Well-being	Good Quality of Life	5	10.0
	Poor Quality of Life	45	90.0
Social Function	Good Quality of Life	4	8.0
	Poor Quality of Life	46	92.0
Pain	Good Quality of Life	6	12.0
	Poor Quality of Life	44	88.0
General Health	Good Quality of Life	9	18.0
	Poor Quality of Life	41	82.0
Total		50	100.0
Healthy Individuals Responses (HI)		Frequency	Percent
Physical Function	Good Quality of Life	40	80.0
	Poor Quality of Life	10	20.0
Physical Health	Good Quality of Life	42	84.0
	Poor Quality of Life	8	16.0
Emotional Problem	Good Quality of Life	44	88.0
	Poor Quality of Life	6	12.0
Energy Fatigue	Good Quality of Life	33	66.0
	Poor Quality of Life	17	34.0
Emotionally Well-being	Good Quality of Life	28	56.0
	Poor Quality of Life	22	44.0
Social Function	Good Quality of Life	35	70.0
	Poor Quality of Life	15	30.0
Pain	Good Quality of Life	34	68.0
	Poor Quality of Life	16	32.0
General Health	Good Quality of Life	38	76.0
	Poor Quality of Life	12	24.0
Total		50	100.0

According to Table 3, the study's statistical analysis revealed that the mean for healthy individuals in the physical function domain was 86.3 while the mean for hemodialysis patients was 34.1. For the physical health domain, the mean was 83.5 for healthy individuals and only 23 for hemodialysis patients. In the emotional issues sector, the mean for healthy individuals was 88.62 while hemodialysis patients had a mean of 44.58. The average energy/fatigue score for healthy people is 63.2 while hemodialysis patients have a score of 39.7. Furthermore, healthy individuals had a mean of 72.32, whereas hemodialysis patients had a mean of 44.32 in the emotional well-being area. In the social function domain, the mean was 81.86 for healthy individuals and 42.28 for hemodialysis patients. Furthermore, the pain domain measured 73.3 for healthy people and 27.5 for hemodialysis patients. Finally, the overall health domain mean for healthy people was 74.82, but for hemodialysis patients, it was 34.78. There was a statistically significant relationship between healthy individuals and hemodialysis patients in two domains: emotional problems (P=0.044) and energy/fatigue (P=0.002).

**Table 3:** Statistics of the short form 36 (SF-36) eight domains of the Medical Outcomes Study HD: hemodialysis; HI: healthy individual

Scale	Items	HI Mean	HD Mean	HI SD	HD SD	P Value
Physical Function	10	86.30	34.10	19.21	32.63	0.234
Physical Health	4	83.50	23.00	31.78	36.71	0.195
Emotional Problem	3	88.62	44.58	28.32	46.44	0.044
Energy/Fatigue	4	63.20	39.70	20.37	16.73	0.002
Emotionally Well-being	5	72.32	44.32	20.16	14.89	1.299
Social Function	2	81.86	42.28	25.25	25.71	1.863
Pain	2	73.30	27.50	32.75	25.31	1.015
General Health	5	74.82	34.78	24.49	17.51	0.524

## Discussion

The findings revealed that patients with HD had a lower quality of life than the control group. According to research conducted by Jalal in 2022 [10] and Ajeebi A in 2020, the majority of patients were male and aged 40 to 50. The majority of respondents in this study were married, which is consistent with the findings of Ajeebi A's study published in 2020 [11]. In addition to these QoL challenges, patients also face various complications associated with HD, complications connected with HD have a broad influence on individuals. Evaluating HD patients' quality of life will aid in determining the quality of care and the effectiveness of medical treatment. As expected, HD patients' quality of life was low in all dimensions, with physical and emotional health being one of the study's most important findings. According to Table 3, there was a statistically significant association between healthy people and hemodialysis patients in two domains: affective problems and energy/fatigue, which is consistent with a study conducted by Birmelé in 2012 [12]. In contrast, in a 2001 study done by Leplège, they obtained higher marks [13]. This could be due to a different population or sample size, and we recommend all hemodialysis centers focus more on these domains with their patients and test them in a much more controlled setting with a larger grid. To improve patients' well-being and quality of life, required interventions, such as counseling, must also be implemented. Infection is the most common consequence among hemodialysis patients, as seen in Figure 2 of the current study. Sahli discovered similar findings in 2017 [14]. When evaluating catheter-related infections, it is critical to examine the key risk factors, such as long-term catheter use and diabetes. To reduce these dangers, we encourage researchers to collaborate with physicians to develop infection control methods that educate and train medical professionals after training for standard infection control such as hand hygiene, personal protective equipment, suitable patient positioning, cleaning and disinfection of patient care equipment, management of fabrics and laundry, safe injection techniques, and proper disposal of needles and other sharp instruments. Hypotension is a medical disorder that results from an inadequate physiological reaction to a reduction in intravascular volume produced by the fast evacuation of a large amount of fluid. This syndrome may appear as an extra consequence following an infection. This disorder has the potential to cause significant health difficulties, such as myocardial infarction, hospitalization due to heart failure or excessive fluid retention, and serious adverse cardiac events such as myocardial infarction, stroke, or cardiovascular-related death. Idiopathic thrombocytopenia is associated with an increased risk of vascular access thrombosis. According to the findings of Ajeebi A's (2020) study [11], more than half of the patients began hemodialysis treatment more than a year ago. The SF-36 subscales yielded consistently poor scores. This observation is similar to the findings of the Bakarman study published in 2019 [15]. This phenomenon is most likely caused by the severity of their symptoms and the frequency with which they receive dialysis. The idea of health-related quality of life has several dimensions, including physical and mental health and social well-being. The assessment and evaluation of a program meant to manage HD patients is dependent on a key indicator. It also plays an important role in improving the health literacy of people with HD, which has an impact on their overall results. The fundamental goal of the suggested healthcare plan for people with chronic kidney disease (CKD) has shifted from increasing survival rates to achieving a decent level of quality of life. The current study had certain drawbacks. Despite effectively establishing connections between numerous parameters and the QoL of patients with CKD, the cross-sectional design of our study limited our ability to undertake longitudinal follow-ups with the patients. As a result, we were unable to identify the exact causes that could have worsened their situation. Furthermore, the data were gathered from a single hospital; a bigger network and a more controlled environment are required the findings provide valuable insights, but they may not fully represent the broader population of hemodialysis patients. This may have been investigated through focus group talks; however, we were unable to discover the cause of low QoL from the patients' perspective. This research could be reproduced as a qualitative study with in-depth interviews or as a prospective study.

## Conclusions

The QoL subscales indicated impairment in HD patients when compared to healthy individuals of the same age and social background in all categories. The average QoL score for HD patients was lower than that of healthy individuals. There was a statistically significant relationship between healthy people and hemodialysis patients in two domains: emotional problems and energy/fatigue levels. Future studies could address these limitations by including a more diverse sample and exploring qualitative aspects of patient experiences.
